# Use of Melatonin in Cancer Treatment: Where Are We?

**DOI:** 10.3390/ijms23073779

**Published:** 2022-03-29

**Authors:** Leilei Wang, Chuan Wang, Wing Shan Choi

**Affiliations:** 1 Division of Oral and Maxillofacial Surgery, Faculty of Dentistry, The University of Hong Kong, Hong Kong SAR, China; lei0302@hku.hk; 2 Division of Periodontology & Implant Dentistry, Faculty of Dentistry, The University of Hong Kong, Hong Kong SAR, China; chuan525@connect.hku.hk

**Keywords:** anticancer, melatonin, molecular mechanisms, reactive oxygen species (ROS), combination therapy

## Abstract

Cancer represents a large group of diseases accounting for nearly 10 million deaths each year. Various treatment strategies, including surgical resection combined with chemotherapy, radiotherapy, and immunotherapy, have been applied for cancer treatment. However, the outcomes remain largely unsatisfying. Melatonin, as an endogenous hormone, is associated with the circadian rhythm moderation. Many physiological functions of melatonin besides sleep–wake cycle control have been identified, such as antioxidant, immunomodulation, and anti-inflammation. In recent years, an increasing number of studies have described the anticancer effects of melatonin. This has drawn our attention to the potential usage of melatonin for cancer treatment in the clinical setting, although huge obstacles still exist before its wide clinical administration is accepted. The exact mechanisms behind its anticancer effects remain unclear, and the specific characters impede its in vivo investigation. In this review, we will summarize the latest advances in melatonin studies, including its chemical properties, the possible mechanisms for its anticancer effects, and the ongoing clinical trials. Importantly, challenges for the clinical application of melatonin will be discussed, accompanied with our perspectives on its future development. Finally, obstacles and perspectives of using melatonin for cancer treatment will be proposed. The present article will provide a comprehensive foundation for applying melatonin as a preventive and therapeutic agent for cancer treatment.

## 1. Introduction

Cancer is the leading cause of death all over the world and its incidence and mortality are rapidly growing worldwide. It has been reported that there were 19.3 million new cases of cancer and almost 10 million deaths from cancer in 2020 [1]. Hence, developing novel prevention and treatment methods is a top priority for reducing the future burden and saving those suffering from cancer. Currently, the most commonly applied method is combined therapy, such as surgery combined with chemotherapy and/or radiation therapy. However, the low 5-year survival rate and poor prognosis are far from satisfactory.

The 2017 Nobel Prize in Physiology or Medicine was awarded to the team identifying the essential role of the circadian rhythm for life health, inspiring us to develop anticancer drugs based on our ‘inner clock’. Melatonin, an endogenous hormone controlling the sleep–wake cycle, came to the attention of scientists for its anticancer effects. Melatonin exerts a variety of biological functions, and numerous studies have identified its anticancer effects as well as the underlying mechanisms. However, the distinct biosynthesis model and the specific modulation manner impede its in vivo investigation and clinical application.

In this review, we comprehensively present the synthesis, metabolism, and physiological and pathological functions of melatonin. Moreover, we explored the anticancer effects of melatonin from three aspects: the molecular mechanisms, the modulation effects on immune cells, and the ROS metabolism. Clinical trials on the anticancer effects of melatonin were reviewed and the synergistic effects of melatonin with conventional anticancer treatment including chemotherapy, radiotherapy, and immunotherapy were also elucidated. Furthermore, we discussed the problems we are facing when using melatonin as an anticancer drug from four aspects. Hopefully, this review will inspire us to establish standard investigation and application approaches of melatonin for cancer treatment.

## 2. Basic Biology of Melatonin

### 2.1. Biosynthesis and Secretion of Melatonin

Melatonin (N-acetyl-5-methoxy-tryptamine) was first discovered in the bovine pineal gland by Lerner in 1958 [2]. It was reported later to exist in plants, clades of invertebrates, and unicellular organisms including bacteria [3,4,5]. Melatonin is mainly secreted by the pineal gland located on the third ventricle of the brain and is synthesized in lymphocytes, bone marrow, eyes, and the gastrointestinal tract [6]. The biosynthesis of melatonin occurs in the mitochondria [7]. Cells firstly obtain amino acid tryptophan from circulation and convert it to serotonin, which is then metabolized into N-acetyl-5-hydroxytryptamine by N-acetyltransferase (AANAT), followed by the transformation to melatonin by hydroxy indole-o-methyltransferase [8]. The rhythmicity of pineal melatonin synthesis is regulated by the ‘master biological clock’, which is located in the hypothalamic suprachiasmatic nucleus (SCN) of all mammals [9]. Light information is received by retinal photoreceptor cells and transferred to the SCN via the optic nerve. The SCN sends this information to the superior cervical ganglion (SCG) through efferent neurons, which, in turn, project sympathetic nerves to the pineal gland. Lacking light at night stimulates the release of noradrenaline from the sympathetic neurons projecting to the pineal gland, which results in the activation of AANAT and subsequently increases the level of melatonin produced by the pineal gland. The melatonin produced by the pineal gland is released into the cerebrospinal fluid (CSF) and blood vessels.

The level of melatonin in cerebrospinal fluid (CSF) and blood reaches the maximal around 2:00 a.m. to 4:00 a.m. and decreases throughout the daytime. The serum concentration of melatonin varies between 80 and 120 pg/mL at night and drops sharply to 10–20 pg/mL during the daylight hours [10]. Exogenous melatonin has been investigated in multiple clinical trials for its potential use in the treatment of circadian and sleep disorders and has been widely used clinically [11]. When taken orally, melatonin could be degraded rapidly in the liver by cytochrome P450 enzymes CYP1A1 and 1A2 enzymes [12], which accounts for its poor and variable bioavailability. To bypass this first-pass metabolism, alternative administration routes have been explored [13].

Fully understanding the biosynthesis and secretion of melatonin is essential for its in vivo investigation and clinical application. Establishing a standard administration time, dosage, and method could maximize the effects of exogenous melatonin in clinical practice. Due to its huge variation of concentration and specific dissolvability, it remains a challenge to apply this particular hormone in cancer treatment. This will be discussed in Section 6.

### 2.2. Biological Effects and Molecular Mechanisms of Melatonin

Melatonin possesses a broad spectrum of biological effects, such as regulating the circadian rhythms and functioning as a potent antioxidant. Moreover, there is a reciprocal association between melatonin and the immune system: The immune system stimulates the synthesis of melatonin, and, in turn, melatonin has immunoregulatory properties [14]. Furthermore, melatonin also exerts anti-inflammatory effects by reducing tumor necrosis factor alpha (TNF-α), interleukin-2 (IL-2), and interferon-gamma (IFN-γ) levels and by enhancing the amounts of interleukin-4 (IL-4), interleukin-10 (IL-10), and interleukin-27 (IL-27) [15]. An increasing number of studies have identified the regulatory effects of melatonin on tumor management in different stages of cancer, including cancer initiation, progression, and metastasis [16].

These biological effects are linked with an array of molecular mechanisms such as binding to membrane receptors, interacting with cytosolic and nuclear proteins, and scavenging radicals directly. Three distinct classes of putative melatonin receptors were reported: (1) MTNR1A (MT1) and MTNR1B (MT2), two membrane receptors of melatonin belonging to the G-protein-coupled receptors’ superfamily [17]; (2) retinoid orphan receptors (ROR), members of the steroid receptor superfamily, which are thought to be the nuclear receptor of melatonin [18], although this concept remains controversial [19]; and (3) the third melatonin binding site MT3, which is identified as quinone reductase 2 [20]. Activation of either MT1 or MT2 by melatonin results in the decreased cyclic adenosine monophosphate (cAMP) and, thus, leads to the inhibition of protein kinase A (PKA) activity. MT2 also interferes with the formation of cGMP via the inhibition of guanylyl cyclase [21]. MT3 may influence the antioxidant and detoxification enzyme expression and reduce proliferation [22]. It has been reported that membrane melatonin receptors are on almost all cell types including retina, brain, suprachiasmatic nucleus, pituitary gland, ovary, cerebral and peripheral artery, kidney, pancreas, fat, and immune cells [23,24]. All of them are available for melatonin to bind and exert the pleiotropic functions.

In summary, melatonin exhibits various biological effects through distinct mechanisms. A full understanding of these mechanisms could facilitate the precise application of melatonin for different diseases and conditions. Structural modification of melatonin based on its receptors may reduce the unwanted side effects to some extent. Further investigations are highly warranted to achieve these aims.

## 3. Anticancer Effect of Melatonin

### 3.1. Underlying Mechanisms/Signaling Pathways

**Figure 1:**
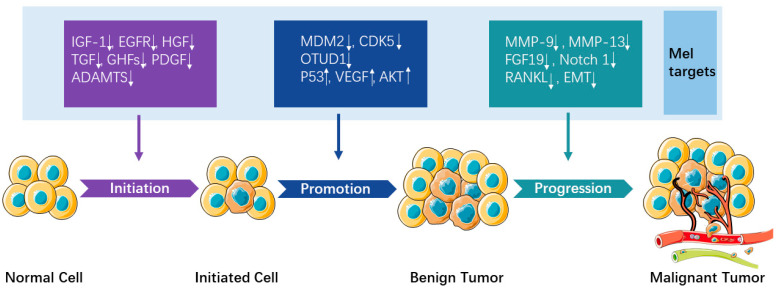
The anticancer effect of melatonin and the involved signaling pathways on tumor initiation, promotion, and progression phases. Up arrows represent upregulation (↑), down arrows represent downregulation (↓).

The anticancer effect of melatonin and the involved signaling pathways were widely reported on tumor initiation, promotion, and progression. In this section, we will summarize the latest evidence on the anticancer effect of melatonin based on literature published from 2019 to 2021 and discuss the findings based on different phases of tumor development (Figure 1), with emphasis on the relationship between melatonin and cancer stem cells (CSCs), microRNA (miRNA), and long non-coding RNAs (lncRNAs).

Initially, studies found that melatonin inhibited the unfavorable tendency of healthy cells to become malignant by downregulating growth factors including prolactin insulin-like growth factor-1 (IGF-1), epidermal growth factor (EGFR), hepatocyte growth factor (HGF), transforming growth factor (TGF), growth hormone-dependent growth factors (GHFs), and platelet-derived growth factors (PDGF) [25]. In an orthotopic xenograft model of renal cell carcinoma, melatonin targeted the post-transcriptional and post-translational modifications of disintegrin and metalloprotease with the thrombospondin motifs’ (ADAMTS) family to retard tumorigenesis [26]. Thus, melatonin helped prevent the initiation of tumor, which is coincident with the clinical observation that people working at night or having low levels of melatonin have a higher risk to get cancer [27].

In the tumor promotion phase, tumor cells are known to downregulate tumor suppressor genes that would trigger the apoptosis pathway, while this process could be rescued by melatonin. For instance, in gastric cancer cells, melatonin induced apoptosis by downregulating the mouse double minute 2 homolog (MDM2) [28]. It also induced apoptosis by suppressing o-GlcNAcylation of cyclin-dependent-like kinase 5 (CDK5) in bladder cancer cells [29]. In a mouse ovarian tumor xenograft model, melatonin prompted apoptosis by stabilizing Bim via Sp1-mediated tumor domain-containing protein 1 (OTUD1) upregulation [30]. In addition, tumor cells are also known to upregulate oncogenes to stimulate growth factors and cell survival signals such as RAS, tumor protein P53 (p53), vascular endothelial growth factor (VEGF), or protein kinase B (AKT). Melatonin suppresses tumor promotion by upregulating p53 [31]. Moreover, melatonin was reported to inhibit cancer growth by downregulating VEGF [32] and AKT [33] in vivo.

In the tumor progression phase, the growth and invasion of tumor cells increase rapidly. Melatonin could inhibit cancer cell growth and downregulate matrix metallopeptidase 9 (MMP-9) [34] and fibroblast growth factor 19 (FGF19) [35] to inhibit cancer cells’ invasion and migration. Moreover, in vivo studies have demonstrated that melatonin alleviates cancer progression by inactivating Notch homolog 1 (Notch 1) receptor [36], suppressing estrogen/ubiquitin C/SDHB-mediated succinate accumulation [37], inhibiting MMP-13 [38], and downregulating receptor activator of nuclear factor kappa-Β ligand (RANKL) expression levels [39]. The epithelial–mesenchymal transition (EMT) is an important process that occurs in the initiation of cancer metastasis. In this process, the epithelial cells lose their cell polarity and cell–cell adhesion, gain migratory and invasive properties to become mesenchymal stem cells, and consequently differentiate into a variety of cell types. It has been reported that melatonin suppresses prostate cancer cells’ migration and invasion by blocking EMT [40]. Moreover, melatonin targets extracellular signal-regulated kinase 1/2 (ERK1/2)-mediated induction of EMT in gallbladder cancer cells [41]. Furthermore, melatonin downregulates the metastasis of colon cancer cells by inhibiting transmembrane protease and serine 4 (TMPRSS4)-mediated EMT [42].

CSCs are cancer cells that can give rise to all cell types found in a particular cancer sample. They may cause relapse and metastasis. Therefore, it is important to develop new therapies targeting CSCs to improve the survival rate and life quality of cancer patients. Recent studies have focused on the anticancer effects of melatonin on CSCs. For instance, in vitro studies showed that by inhibiting phospholipase C (PLC), ERK/p38, and β-catenin and Twist signaling pathways, melatonin exerts the potential to suppress lung cancer stemness [43]. With regard to head and neck squamous cell carcinoma, melatonin combined with verteporfin induced apoptosis of CSCs by regulating mitochondrial function via targeting Parkin/TOM20 [44]. As for breast CSCs, melatonin had significant inhibitory effect on hypoxia-induced vasculogenic mimicry via suppressing EMT [45]. In addition, a study using mice with melanoma xenografts models showed that melatonin combined with vemurafenib promotes the apoptosis, cell cycle arrest, and weakening of stemness in melanoma cells via the iNOS/hTERT signaling pathway [46].

MiRNA is a small, single-stranded, non-coding RNA molecule that functions in RNA silencing and post-transcriptional regulation. MiRNA is associated with cancer progression, diagnosis, and treatment. The role of melatonin in cancer-related miRNA has been widely investigated in the past 3 years. For instance, miR-155 is responsible for cancer progression, and the expression of miR-155 can be significantly suppressed by melatonin in oral cancer cell lines [47]. After analyzing the interaction between 36 angio-miRNAs and the melatonin-modulated hypoxia-inducible factor-1 (HIF1)/VEGF/MMP9 axis, researchers found that miR-15b, miR-18a-5p, miR-23a-3p, miR-92a-3p, miR-130a-5p, and miR-200b-3p may play important roles in malignant glioblastoma multiforme (GBM) tumorigenesis and invasion and that all of these miRNAs respond to melatonin therapy [48]. An in vivo study demonstrated that, in adenocarcinoma, melatonin exerted abilities of regulating the interplay between forkhead box protein O1 (FoxO1), miR96, and miR215 signaling. This signaling axis may be a novel target for melatonin to diminish cancer cell growth, survival, and metastasis [49]. Melatonin can upregulate miRNA-34a/449a to promote apoptosis in colorectal cancer cells and a xenograft mouse model as well [50].

LncRNA is a type of RNA with more than 200 nucleotides without protein translation. Cancer is a disease associated with numerous genetic mutations and chromosomal translocations. LncRNA plays key roles in regulating gene expression and chromatin dynamics; their detailed characterizations in cancer have been annotated [51]. However, it was not until the past few years that the interactions between melatonin and lncRNA during cancer treatment began to draw people’s attention. For instance, a new melatonin-attenuated lncRNA, melatonin-regulated oral cancer stimulator (MROS-1), has been identified. It mediates protein prune homolog 2 (PRUNE2) expression by activating the JAK-STAT pathway and results in the suppression of oral cancer cell motility [52]. Moreover, in human osteosarcoma cells, melatonin represses the expression of lncRNA JPX by modulating the Wnt/β-catenin signaling pathway, thus inhibiting cancer cells’ viability, proliferation, migration, invasion, and metastasis [53]. Furthermore, in a mouse xenograft model, melatonin inhibited triple-negative breast cancer progression through the Inc049808-FUN14 domain-containing 1 (FUNDC1) pathway [54].

Overall, melatonin exerts its anticancer functions during tumor initiation, promotion, and progression by targeting various signaling pathways. The increasingly reported regulation effects of melatonin on CSCs, miRNA, and lncRNA during cancer treatment provide us new insights for cancer management. Nevertheless, melatonin as an endogenous hormone could regulate almost all the cell types. Other systems such as the immune system may also play important roles in melatonin-regulated cancer suppression.

### 3.2. Modulation of Immune Responses by Melatonin

The tumor microenvironment (TME) is infiltrated by various immune cells. The immune system within the TME has pivotal roles in both inhibition and progression of cancer cells. Natural killer (NK) cells and cytotoxic T lymphocytes (CTLs) are the main oncostatic immune cells, while T regulatory cells (Tregs) and cancer-associated fibroblasts (CAFs) facilitate immune escape of cancer cells. Moreover, tumor-associated macrophages (TAMs) release cytokines that attenuate the activity of the immune system against tumor cells or stimulate angiogenesis [55].

Melatonin exhibits its anticancer effects by modulating the immune system and shifting the immune response toward cancer cells in the TME [56]. For instance, T cells express membrane receptors and nuclear binding sites of melatonin, which could modulate activation and differentiation of T cells. Melatonin stimulates the proliferation of CTLs by increasing the expression level of inflammation cytokines such as IFN-γ, TNF-α, and IL-6 [57]. Melatonin has also been demonstrated to increase the number and activity of CD8+ cytotoxic T cells [58].

Several studies described the effect of melatonin on Tregs. Melatonin can suppress TGF-β and IL-4 (inducer of Tregs) and upregulate IFN-γ (inhibitor of Tregs) to modulate Tregs’ activity. An animal study showed that 100 mg/kg of melatonin can significantly downregulate the Tregs’ ratio in a tumor [59]. Patients with an untreatable, metastatic, solid tumor have shown a reduction of Tregs [60].

Melatonin could also increase the infiltration activity of NK cells [61]. An animal study showed that a supplement with melatonin augmented NK cell numbers and extended its survival time [62]. Melatonin also increased the IL-2 secretion by upregulating the MT1 receptor, increasing NK cell numbers [63].

Macrophages also have membrane receptors for melatonin [64]. Notably, melatonin modulates macrophage polarization through various pathways such as NF-κB [65]; whether melatonin shifts a macrophage from M1 to M2 or vice versa depends on the environmental condition. Moreover, miRNAs and mitochondrial dynamics also participate in the process of macrophage polarization caused by melatonin [66]. Since the M1-type macrophage exhibits an anticancer effect, melatonin could facilitate cancer management by modulating the M1 macrophage response.

The direct effect of melatonin on CAFs has been explored. For instance, melatonin indirectly decreases gastric cancer cell proliferation and invasion by inhibiting the production of MMP-2 and MMP-9 in CAFs [67]. TGF-β is one of the most potent immune suppressive cytokines, and CAFs are one of the main cells for the release of TGF-β. Since melatonin has been proven to suppress the expression level of TGF-β [68], it is reasonable to speculate that suppressing TGF-β secretion and weakening activities of CAFs are among the underlying mechanisms for the anticancer effects of melatonin.

Indeed, increasing evidence has shown that inflammation and the immune system play important roles in cancer progression. Recently, the paradigm of cancer research has shifted from cancer cell to TME. Numerous studies have proven that melatonin could regulate the immune cells in TEM. Thus, melatonin may function as an alternative approach for immune-regulation therapy.

### 3.3. Relationship between Melatonin, ROS Metabolism, and Tumor Progression

Oxidative stress caused by ROS is involved in the etiology of many diseases, such as cardiovascular diseases and cancer. It facilitates cancer development by inducing DNA oxidation and genomic instability [69]. Melatonin acts as a powerful antioxidant by extinguishing ROS through its metabolites such as N (1)-acetyl-5-methoxykynuramine (AMK), cyclic-3-hydroxymelatonin (cyclic-3OHM), and 6-hydroxymelatonin (6-OHmel) [70]. However, a high concentration (µM to mM) of melatonin could increase the ROS level, leading to induced cytotoxicity in certain tumor cell lines [71].

To explain why melatonin, as an antioxidant, can increase ROS levels in certain types of tumor cells, it is necessary to understand the glucose metabolism (glycolysis) and cellular respiration (oxidative phosphorylation (OXPHOS)) in the inner mitochondrial membrane.

Cancer cell metabolism is different from healthy cells [72]. To support the rapid proliferation, cancer cells have to modify their metabolism to adapt to the nutrient-deprived environments. Changes in metabolism include glycolysis, gluconeogenesis, glutaminolysis, pentose phosphate pathway (PPP), mitochondrial biogenesis, and lipid metabolism, which all contribute to tumor development, invasion, and metastasis [73]. In normal cells, pyruvate generated from the glycolysis process is transported to the mitochondria and converted into acetyl coenzyme A (acetyl-CoA); however, in most tumor cells, pyruvate is metabolized into lactate in the cytosol. Thus, the generation of acetyl-CoA is interrupted. The Warburg effect is the process that restricts pyruvate from being transported to the mitochondria but allows glucose to be metabolized into pyruvate in the cytosol, facilitating cancer cells’ rapid proliferation, avoiding apoptosis and enhancing the invasion and metastasis. In addition, as an absolute requirement in the TCA cycle and OXPHOS, the decrease of acetyl-CoA will compromise the OXPHOS, which in turn reduces ROS generation [74]. This change in tumor metabolic activities is also beneficial to cancer cells’ growth. As we described above, AANAT is essential for melatonin synthesis. Acetyl-CoA, as an acetyl donor of AANAT, is equally required for melatonin synthesis. Therefore, we may conclude that cancer cells prevent the formation of melatonin. In conclusion, the changes in cancer cell metabolism make it express a higher level of ROS and lower level of melatonin compared to normal cells.

There are two possible mechanisms for the increase of the ROS level by melatonin cancer cells: (1) Melatonin directly stimulates the ROS and (2) melatonin breaks the cellular redox balance by decreasing the number of molecules that eliminate ROS. The first mechanism could occur when melatonin activates electron transport chain complexes. Some important reductive molecules such as NADPH/NADH that are required for glutathione and thioredoxin to work as antioxidants are mostly produced during glycolysis and the TCA cycle. During this progress, electrons are carried by electron transporters and finally bind to oxygen to form water. These events, termed as the electron transport chain (ETC), contain electron transporters that are distributed into four enzyme complexes: complex I (NADH ubiquinone reductase), complex II (succinate ubiquinone reductase), complex III (ubiquinol–cytochrome c reductase), and complex IV (cytochrome c oxidase). Electrons that evade the ETC could generate free radicals. Therefore, ROS could be produced by complexes I and III. Studies have demonstrated that melatonin is able to modulate ROS formation [75,76]. Interestingly, melatonin in pharmacological doses induces the accumulation of ROS in cancer cells by causing the allosteric modulation of complex III. This activity is more obvious in cancer cells than in normal cells and can trigger the activation of the pro-apoptotic pathway [77]. Based on the above knowledge, the second mechanism may be effective if melatonin reduces NADPH/NADH by altering the tumor metabolic activities. However, further investigations are required to confirm this mechanism.

Recently, numerous studies have described the role of melatonin on ROS metabolism and its anticancer potential. In oral squamous cell carcinomas (OSCCs), melatonin enhances ROS production and DNA damage to prevent OSCCs’ initiation and progression [78]. Similar functions were verified in colorectal cancer [79] and breast cancer [80]. These two studies further illustrate that ROS-mediated endoplasmic reticulum stress (EnR) is involved in the apoptosis caused by ROS overaccumulation.

Above all, the distinct metabolism in cancer cells produces less melatonin and higher levels of ROS than normal cells. Exogenous melatonin reduces ROS in normal cells and exerts its anticancer effects by generating more ROS in cancer cells. Further investigations are needed to verify the dual function of melatonin in ROS production to establish standard administration approaches in different clinical practices.

## 4. Clinical Trial Based on the Anticancer Effects of Melatonin

Multiple in vivo and in vitro studies have been carried out and confirmed the beneficial effects of melatonin in various cancers. To further facilitate melatonin to be used as an adjuvant for traditional anticancer therapy, researchers have investigated the effectiveness of melatonin in clinical studies and patients (Table 1). An epidemiology report showed that people working at night or people having low levels of melatonin have a higher risk to get cancer [27]. Since then, researchers have studied the relationship between melatonin and cancer. Almost all the clinical studies used melatonin in combination with chemotherapy or as protective therapy, and they showed that application of melatonin led to positive impacts on the anticancer treatment, including alleviating the chemotherapy-related side effects, reducing the incidence of depressive symptoms, and improving the quality of sleep of patients with cancer [81].

In a randomized, double-blind, clinical study, melatonin was co-administered to patients with head and neck cancer. Results showed that not only could melatonin inhibit the antioxidant capacity of the patients but it also reduced mucositis and ameliorated pain [82,83]. In addition, combining melatonin with a cisplatin-based standard treatment reduced anemia, a common side effect of cisplatin [84]. Moreover, the effect of melatonin in ameliorating the side effects of chemotherapy was also investigated in patients with gastrointestinal cancer, showing that melatonin could maintain the body weight but failed to attenuate cachexia [85]. A clinical study based on metastatic colorectal patients revealed that the group that received low-dose, subcutaneous IL-2 with melatonin after a first-line therapy of 5-FU had a higher percentage of survival at 1 year compared to those who only received 5-FU treatment, which suggested that the low-dose, subcutaneous IL-2 and melatonin can be used as a second-line therapy for colon cancer therapy [86].

A randomized clinical trial showed that metastatic breast cancer patients who received the treatment with both tamoxifen and melatonin had a higher relative response compared with those receiving tamoxifen alone [87]. Conversely, some clinical trials showed contradictory results. Postmenopausal breast cancer patients (stages 0–III) who had completed anticancer treatment and received an oral supplement of melatonin (3 mg/day for 4 months) did not significantly improve the levels of serum biomarkers (estradiol, IGF1, IGFBP-3) related to breast cancer [88].

The use of melatonin in non-small cell lung cancer (NSCLC) patients was also investigated, showing that melatonin improved the quality of life but did not demonstrate any protective effects against chemotherapy-related side effects [89]. Moreover, adjuvant melatonin following resection of NSCLC increased the 2-year disease-free survival (DFS) in patients with late-stage disease but did not exhibit beneficial effects in quality of life, symptoms, or immune function [90]. Further studies are needed to explore the beneficial effects of melatonin on this leading cause of death from cancer worldwide.

Although a great deal of clinical evidence has confirmed the anticancer effects of melatonin, some conflicting results also exist. More in vivo studies and clinical trials are needed to establish its proper clinical application in cancer treatment.

## 5. Melatonin in Combination with Other Anticancer Therapies

Despite significant advancements in the development of anticancer therapy, chemotherapy remains one of the most common cancer treatments. The main problems of chemotherapy are the adverse side effects to many organs and systems, as well as the rising occurrence of medication resistance. Many benefits of melatonin applied as an adjuvant to chemotherapy have been reported (Table 2), including enhancing the drug efficiency [91,92,93] and alleviating the side effects [94]. Melatonin has been reported to sensitize cancer cells to chemotherapy via promoting apoptosis [95,96] and autophagy in cancer cells [97]. Interestingly, one study showed that apoptosis in human breast cancer cells caused by melatonin and doxorubicin was enhanced via the autophagy-dependent reduction in AMPKα1 transcription [98].

The combination of melatonin with radiotherapy could also provide synergistic antitumoral outcomes and relieve drug resistance. For example, a study showed that melatonin counteracted the inhibitory effects of radiation on the differentiation of pre-adipocytes by stimulating C/EBPα (CCAAT/enhancer binding protein alpha) and PPARγ (peroxisome proliferator-activated receptor gamma) expression [99]. In vitro studies have proven that the use of melatonin could enhance the effect of radiotherapy, while investigations on human subjects are scarce. The combination therapy of melatonin and 60 Gy of radiation in patients with glioblastomas was reported to increase the 1-year survival rate and improve life quality [100], while no clinical value was found in patients with cerebral metastases who were administered 30 Gy of radiotherapy and 20 mg of melatonin [101].

A cancer vaccine is a vaccine that either treats an existing cancer or impedes cancer development. Cancer cells arise routinely and could be destroyed by the immune system; if not, a tumor forms [102]. Some types of cancer are caused by viruses, such as cervical cancer and liver cancer, which could be prevented by the human papillomavirus (HPV) vaccine and hepatitis B vaccine, respectively. Some types of cancer are caused by a bacterial infection, such as stomach cancer. Therefore, the anti-Helicobacter pylori vaccine can prevent stomach cancer development. These are traditional vaccines. Another type of cancer vaccine separates proteins from cancer cells and targets these proteins, which act as antigens to boost the immune system to kill cancer cells. Melatonin has the potential to stimulate the immune system; thus, it may have a synergistic effect when combined with cancer vaccination. One in vivo study proved that melatonin enhanced the cancer vaccine efficiency against HPV-associated tumors by inhibiting the expression level of IL-10 and VEGF in the tumor microenvironment of vaccinated mice [103]. Another study showed that the combined use of melatonin and DL-1MT (indoleamine 2,3-dioxygenase-1 inhibitor) enhanced vaccine-induced protective cellular immunity to hpv16-associated tumors [104].

Immunotherapy is a cancer treatment that works by boosting the immune system’s activity. It can be achieved by activating NK cells and CD8^+^ T lymphocytes or by inhibiting TAMs, Tregs, and immune checkpoints [105]. Antitumor immunotherapy has attracted increasing attention recently. CTLA4, PD-1, and PD-L1 are the most acknowledged targets for immune checkpoint inhibitors. Inhibition of these immune checkpoints is known to elicit antitumor responses. However, the response rate is quite low [106]. Combination therapy may be a way out of this dilemma. Melatonin has an immunomodulation effect, as described above, and it has been investigated as an adjuvant with immunotherapy drugs for a long time. Patients with advanced solid tumors can benefit from melatonin as an antitumor agent, which increases the effectiveness of IL-2 [107]. Other studies further verified that melatonin was able to improve the efficiency of immunotherapies by stimulating lymphocyte proliferation [108] or by suppressing the yes-associated protein (YAP)/PD-L1 axis and enhancing antitumor immunity [109]. Moreover, the administration of melatonin (100 mg/day) in conjunction with anti-PD-1 led to a better prognosis among patients with various cancers [110]. Thus, it is promising for melatonin to be used as an adjunct to immunotherapy.

**Table 2:** Melatonin in combination with other anticancer therapies.

Combination Therapy	Melatonin Effect	Mechanisms of Action	Reference
Mel + chemotherapy	Induction of endoplasmic reticulum stress and apoptosis	Inhibition of cellular PrPC	[91]
	Inhibition of cell growth; induction of apoptosis	Upregulation of miR-215-5p and a concomitant downregulation of TYMS	[92]
	Enhancing the cytotoxic effects	Destruction of HER2 protein	[93]
	Inhibition of cell proliferation, invasion, and migration; induction of apoptosis	Regulating EZH2 expression	[96]
	Suppressing autophagy	Inhibition of NR4A1, CTSL, and Atg12	[97]
	Induction of apoptosis and autophagy	Decreasing AMPK α1 expression	[98]
Mel + radiotherapy	Counteracting the inhibitory effect of radiation on preadipocytes’ differentiation	Increasing C/EBPα, PPARγ expression; decreasing TNFα expression	[99]
	Prolonging the survival time; improving the quality of life of patients	Reducing radiotherapy-related toxicities	[100]
Mel + cancer vaccination	Enhancing cancer vaccine efficiency	Inhibiting IL-10 and VEGF expression level	[103]
	Enhancing the antitumor protective immunity of vaccine	Improving the numbers of circulating E7-specific CD8+ T cells in mice	[104]
Mel + immunotherapy	Enhancing IL-2 antitumor immune effect	Increasing the susceptibility of cancer cells to the cytolysis	[107]
	Improving the immune response	Stimulating lymphocyte proliferation; inhibiting macrophage-induced inflammatory status	[110]

## 6. Problems with Using Melatonin as an Anticancer Drug

### 6.1. Chemical Properties of Melatonin

The half-life of melatonin by both oral and intravenous routes is approximately 45 min (28–126 min), and the oral bioavailability is low with significant individual variability (9–33%) [111]. Melatonin is widely used clinically for the short-term management of sleep disorders, such as insomnia due to jet lag or shift work. It is typically taken orally at a dose ranging from 3 mg to 10 mg daily; for cancer treatment, the dosage of melatonin would be much higher. Consequently, the risk and severity of the side effects may increase. Therefore, it is necessary to search for an alternative administration route to improve the bioavailability so as to establish the optimal dosage for cancer treatment.

In order to use melatonin for cancer treatment, the drug form has to be stable with a reasonable length of shelf life so as to ensure reliable drug delivery. However, melatonin has low water solubility and high permeability. Many trials have been attempted to look for the right solvent for melatonin and test for its stability over time. For instance, 100–113 µg of melatonin dissolved in 5% ethanol and 95% isotonic saline remained stable for 6 months, while 50 µg/mL of melatonin dissolved in phosphate buffer degraded over 21 days at various pH ranges [112,113]. When 10 mg/mL melatonin was dissolved in dimethyl sulfoxide (DMSO), it showed stability for over 45 days [114].

Since melatonin has low water solubility and poor chemical stability in an organic solvent, novel, biocompatible vehicles need to be developed to improve the bioavailability and solubility of melatonin. Nanosized carriers have aroused great interest for their unique properties including increased therapeutic efficacy, reduced side effects, and improved life quality of patients [115]. Nanoparticles have been used to load melatonin for cancer treatment. An Fe_3_O_4_ nanoparticle, as a magnetic nanocarrier for the co-delivery of doxorubicin (DOX) with melatonin, enhanced apoptosis of osteosarcoma cells. DOX combined with melatonin had a synergistic antitumor effect as well, especially in a nano-formulation form [116]. Moreover, nanostructured lipid carriers (NLCs) loaded with melatonin could release melatonin sustainably and improve the efficacy of tamoxifen in breast cancer treatment [117]. Furthermore, chitosan-tripolyphosphate nanoparticles loaded with melatonin were proven to attenuate etoposide-induced genotoxicity in HepG2 cells by decreasing the intracellular ROS [118]. In addition, melatonin was loaded in 3D-printed magnesium-polycaprolactone to treat osteosarcoma (OS), with a high efficiency in inhibiting OS cells’ proliferation, invasion, and metastasis both in vitro and in vivo [119]. Currently, the two most popular forms of melatonin are tablets and capsules, and the concept of developing nanoparticles for melatonin administration is still in its early stages, with studies focused on in vitro delivery on diverse human conditions [120].

### 6.2. The Measurement of Melatonin

Numerous beneficial roles of melatonin in modulating circadian rhythms and related disease have been reported. Hence, melatonin levels under various clinical and physiological conditions are important index values. However, how to precisely measure the melatonin concentration remains a huge challenge.

The fundamental problem is which sample should be collected: plasma, saliva, or urine. Endocrinology studies normally take blood samples to test for hormone levels and to assess the function of relevant glands. Since melatonin is secreted directly into the bloodstream, the measurement of melatonin in blood plasma is the most direct way. Melatonin levels in plasma are expected to be less than 5 pg/mL during the daytime [121]. Therefore, the melatonin assay kits should have high sensitivity. Moreover, melatonin could also be found in saliva at night. Studies subsequently proved the correlation between plasma and saliva melatonin levels [122], despite that the use of salivary melatonin to speculate on serum concentration was once thought to be non-convincing [123]. Furthermore, testing the 6-sulphatoxymelatonin level in urine is an accurate method to reflect the pineal melatonin [124]. However, the commercial kits for urinary 6-sulphatoxymelatonin measurement could seldom achieve the standards set by mass spectrometry assays.

Another important factor for obtaining an accurate melatonin measurement is the sample collection time. It is essential to collect multiple samples at different time points, especially when exploring the onset of melatonin secretion or the total output of the pineal gland. The melatonin level in venous blood plasma remains low during the daytime, increases sharply at night, reaching the maximum at around 3:00 a.m., and then declines to low levels at around 7:00 a.m. to 8:00 a.m. in most people. Most animals also share a similar phenomenon that the melatonin level is low during the daytime and high at night.

It is essential to select an appropriate assay kit for the test. Numerous assay kits have been developed since the 1970s to measure melatonin levels in blood and saliva. It is suggested that the commercial assays should be sensitive enough to detect low concentrations (<2 pg/mL) [125]. Unfortunately, very few kits available on the market could achieve such high sensitivity, thus leading to inaccurate measurements.

In short, the measurement of the melatonin level is often required in melatonin studies. Researchers have to pay attention to three pertinent aspects in the study design: which sample to collect, sample collection time, and the selection of assay kits. Neglecting these issues always leads to huge variations among different studies, inaccurate data, and an incorrect conclusion. This may provide explanation to the conflicting results in the literature, which hinders the determination of the therapeutic level of melatonin in cancer treatment.

### 6.3. The Safety Profile and Dosing of Melatonin

Melatonin is well acknowledged as a drug with no significant toxicity both in physiological and pharmacological concentrations. Nonetheless, we cannot draw this conclusion without elucidating the dose and duration of melatonin treatment. Researchers have yet to determine the optimal dosage of melatonin for adjuvant cancer therapy or cancer prevention. A systematic review compared 50 studies with melatonin administration ranging from 0.3 mg–1600 mg daily for 4 weeks to 3.5 years (15 ± months, average). Twenty-six studies reported no adverse events. The reported adverse events were mostly related to psychomotor and neurocognitive dysfunctions, fatigue, and excessive sleepiness [126], which could be avoided by taking melatonin at night and using the proper dosage with short-term administration. However, when applied for cancer treatment, relatively high doses both in vitro (0.1–10 mM) and in vivo (5 mg–200 mg/d) and long-term duration are needed, which may cause severe side effects. Moreover, cancer patients have a pathological condition and they are receiving other ongoing anticancer treatments. The fragile body condition may exacerbate the side effects.

In summary, it is vital to identify the possible side effects of melatonin when used in cancer treatment. In the literature, most of the studies reported a low incidence of adverse events but the melatonin was administered at a low dose and for a short duration. In view of the pathological status and possible concurrent treatment in cancer patients, further investigations are warranted to verify the safety profile and dosage of melatonin in cancer treatment.

### 6.4. As a Single Anticancer Drug or Adjuvant Anticancer Drug?

Current evidence suggests melatonin to be used as an adjuvant or protective therapy. As an adjuvant therapy, it can increase the treatment efficacy; as a protective therapy, it can ameliorate adverse side effects of chemo- and radiotherapy during cancer treatment [127,128].

Although melatonin has high efficacy and low toxicity in cancer treatment, very few companies want to develop this hormone, per se, as it is a natural and nonpatentable molecule. Moreover, despite numerous studies that have demonstrated the anticancer effects of melatonin, some studies also showed that the use of melatonin alone exerted less or even no effect on reducing cancer markers [88]. Some epidemiological studies connecting the body’s circadian melatonin levels to cancer incidence concluded either significant or no associations [129,130,131]. Melatonin exerted anti-proliferative and anti-migrating effects on a melanoma cell line, but similar effects could not be demonstrated in vivo [132]. These inconsistent results also occurred in patients undergoing chemotherapy. A prospective cohort study investigating breast cancer patients undergoing chemotherapy showed that the repeated administration of chemotherapy dampened nocturnal melatonin production, which caused sleep disturbance and sleep–wake activity rhythm disruption, suggesting that the supplementation of melatonin may improve the quality of life [133]. However, this potential benefit of melatonin was not demonstrated in melanoma patients receiving dacarbazine [134].

All in all, the existing studies support the use of melatonin as an adjuvant or protective anticancer drug because they used a relatively low and uniform dosage of melatonin. 

However, when assessing the anticancer efficacy of melatonin alone, the findings from both basic and clinical studies were inconsistent due to varied dosages and times of administration. Further investigations are needed to develop a suitable and uniform administration dosage and time for precise treatment.

## 7. Conclusions and Perspective

Cancer eradication has been an aim for scientists and physicians for a long time. The ideal anticancer therapeutic is to use medications that have already been tested and used in a clinical setting with a good safety profile and that are efficacious in treating cancer. Melatonin, a pleiotropic hormone used in treating sleep disorders, has minimal toxicity and could mitigate cancer at the initiation, promotion, and progression phases. Nonetheless, there are four main problems when using melatonin as an anticancer drug: (1) a low biocompatibility of melatonin, (2) a non-uniform measurement of the melatonin level, leading to an uncertain therapeutic level, (3) an uncertain dosing regimen and side effects on cancer patients, and (4) an uncertain administration approach. To facilitate the clinical application of melatonin, some measures need to be taken. Firstly, new drug forms or drug carrier systems should be developed to improve the melatonin bioavailability. Secondly, criteria on the sample collection time and sample sources need to be clarified. Thirdly, more studies are needed to elucidate the optimal dosage and long-term safety of melatonin in cancer patients. Finally, understanding the molecular mechanisms and relevant clinical studies are warranted to provide further evidence for melatonin to be used clinically as an anticancer drug. Considering melatonin’s multiple benefits on human health, we hope this review will help to provide different perspectives for enriching our understanding about the potentials and limitations of melatonin in anticancer therapy. In addition, these findings may facilitate the development of melatonin as a potential alternative for cancer treatment either alone or in combination with other anticancer drugs.

## Figures and Tables

**Table 1:** Clinical evidence for the anticancer effects of melatonin.

Evidence Type	Study Type	Conclusion	Reference
Positive evidence	Epidemiology report	People working at night or people having low levels of melatonin have higher risk to get cancer.	[27]
	Prospective phase II trial	Melatonin is a potentially useful therapeutic agent for improving sleep and quality of life in cancer patients.	[81]
	Randomized, phase II clinical trial	Melatonin reduced mucositis and ameliorated pain in patients treated with concurrent radiation.	[82,83]
	Randomized clinical trial	Concomitant administration of melatonin may reduce cisplatin-induced anemia in cancer patients.	[84]
	Randomized pilot study	Melatonin may produce a weight-stabilizing effect.	[85]
	Randomized study	Low-dose, subcutaneous IL-2 and melatonin can be used as a second-line therapy for colon cancer therapy.	[86]
	Pilot phase II study	Melatonin induced objective tumor regressions in metastatic breast cancer patients refractory to tamoxifen alone.	[87]
Negative evidence	Randomized, controlled trial	Short-term melatonin treatment did not influence the serum biomarkers related to breast cancer.	[88]
	Randomized, double-blind, placebo-controlled study	Melatonin in combination with chemotherapy did not affect survival and adverse events of advanced patients with NSCLC.	[89]
	Randomized, placebo-controlled clinical trial	Melatonin did not exhibit beneficial effects in quality of life, symptoms, or immune function.	[90]
